# ^1^H, ^13^C, ^15^N backbone and IVL methyl group resonance assignment of the fungal β-glucosidase from *Trichoderma reesei*

**DOI:** 10.1007/s12104-020-09959-2

**Published:** 2020-06-19

**Authors:** Eleni Makraki, Marta G. Carneiro, Alex Heyam, A. B. Eiso, Gregg Siegal, Roderick E. Hubbard

**Affiliations:** 1 YSBL, Department of Chemistry, University of York, Heslington, York UK; 2 ZoBio BV, J.H. Oortweg 19, 2333 CH Leiden, The Netherlands; 3 Division of Medicinal Chemistry, Amsterdam Institute for Molecules, Medicines and Systems (AIMMS), Vrije Universiteit, De Boelelaan 1108, 1081 HZ Amsterdam, Netherlands; 4 Vernalis Research, Granta Park, Abington, Cambridge, UK

**Keywords:** *Tr*Bgl2, β-glucosidase, Resonance assignment, Enzyme activators

## Abstract

β-glucosidases have received considerable attention due to their essential role in bioethanol production from lignocellulosic biomass. β-glucosidase can hydrolyse cellobiose in cellulose degradation and its low activity has been considered as one of the main limiting steps in the process. Large-scale conversions of cellulose therefore require high enzyme concentration which increases the cost. β-glucosidases with improved activity and thermostability are therefore of great commercial interest. The fungus *Trichoderma reseei* expresses thermostable cellulolytic enzymes which have been widely studied as attractive targets for industrial applications. Genetically modified β-glucosidases from *Trichoderma reseei* have been recently commercialised. We have developed an approach in which screening of low molecular weight molecules (fragments) identifies compounds that increase enzyme activity and are currently characterizing fragment-based activators of *Tr*Bgl2. A structural analysis of the 55 kDa apo form of *Tr*Bgl2 revealed a classical (α/β)_8_-TIM barrel fold. In the present study we present a partial assignment of backbone chemical shifts, along with those of the Ile (I)-Val (V)-Leu (L) methyl groups of *Tr*Bgl2. These data will be used to characterize the interaction of *Tr*Bgl2 with the small molecule activators.

## Biological context

The depletion of fossil fuel in combination with the increasing demand for energy worldwide has instigated research on alternative and sustainable energy sources such as biofuels. Lignocellulosic (LC) biomass such as wood, agricultural residues and dedicated energy crops are abundant and available at low cost and have received considerable global attention as the most promising alternative, renewable source for biofuel production. LC biomass, the structural backbone of all plant cell walls, is composed mainly of cellulose, in combination with hemicellulose and lignin (Service 2007). A mixture of enzymes that together are known as cellulase, catalyze cellulose degradation and comprise three categories of enzymes; endoglucanases (EC 3.2.1.4), exoglucanases or cellobiohydrolases (EC 3.2.1.91) and β-glucosidases (EC 3.2.1.21) (Sticklen 2008) (Brethauer and Studer 2015). Endoglucanases cleave the internal β-1,4-glycosidic bonds of cellulose microfibrils releasing small fragments. Subsequently, exoglucanases or cellobiohydrolases (CBH) act on the reducing and non-reducing ends resulting in short chain cello-oligosaccharides such as cellobiose, which are hydrolysed into glucose by the action of β-glucosidases (Sticklen 2008). However, the activity of β-glucosidase is a rate limiting step which results in accumulation of cellobiose and subsequent inhibition of other cellulases (Lynd et al. 2002; Bommarius et al. 2008; Resa and Buckin 2011; Brethauer and Studer 2015). Currently, this is overcome by using high concentrations of β-glucosidases increasing the cost of large-scale conversions. Many efforts have therefore been directed towards the improvement of catalytic activity and thermostability of the enzyme using mainly traditional genetic approaches (Lee et al. 2012).

*Trichoderma reesei* produces large amounts of thermostable cellulolytic enzymes, which make them attractive targets for industrial applications (Gao et al. 2017). Genetically modified β-glucosidases from *Trichoderma reesei* have been commercialized and are included in cellulolytic enzyme cocktails that have been developed by companies. Recently, Jeng et al. (2011) solved the crystal structure of the 55 kDa β-glucosidase from *Trichoderma reesei* (*Tr*Bgl2) at 1.63 Å resolution, which was found to adopt a classical (α/β)_8_-TIM barrel fold and be in tight association with a TRIS molecule at the active site (Jeng et al. 2011).

In previous studies, we have identified fragment-based activators of enzyme activity (Darby et al. 2014). We are currently characterizing fragment-based activators of *Tr*Bgl2. The work reported here is a partial assignment of backbone chemical shifts, along with those of the Ile (I)-Val (V)-Leu (L) methyl groups of *Tr*Bgl2. These assignments will provide the basis for a study of the interaction of *Tr*Bgl2 with the small molecule activators.

## Methods and experiments

### Sample preparation

The codon-optimized DNA sequence for *Tr*Bgl2 was inserted into the pET-YSBLIC3C plasmid with an N-terminal His_6_ cleavable tag (Fogg and Wilkinson 2008). The plasmid was transformed into the E. coli BL21 (DE3) bacterial strain. Expression was performed using the method from Gans et al. (2010) with some modifications. Labelled samples were grown in M9/D_2_O minimal media containing ^15^NH_4_Cl (1.0 g/L) and ^13^C_6_-glucose (1.5 g/L). The cells were incubated at 37 °C until reaching an OD_600_ of 0.4 when 2-Keto-3-(methyl-d3)-butyric acid- 1,2,3,4-13C4, 3-d sodium salt (SIGMA, Cat. 637858) and 2-Ketobutyric acid-13C4,3,3-d2 sodium salt hydrate (SIGMA, Cat. 607541) were added (Gans et al. 2010; Tugarinov and Kay 2003). The cells were further incubated at 37 °C until reaching an OD_600_ of 0.7–0.8. The temperature was then decreased to 16 °C and the protein expression was induced with 0.5 mM IPTG and cells were harvested after overnight incubation at 16 °C. The cells were resuspended in a buffer containing 20 mM TRIS–HCl pH 7.5, 400 mM NaCl, 10 mM Imidazole and then disrupted by using the Cell Disruption System (Benchtop, Constant Systems Ltd). After centrifugation, the soluble *Tr*Bgl2 was initially purified by Ni^2+^-NTA chromatography as described previously (Jeng et al. 2011). The sample was then loaded onto a size exclusion column (16/600 Superdex 200, GE Healthcare), and eluted with a buffer containing 50 mM TRIS–HCl pH 8, 100 mM NaCl, 3 mM DTT. The purified protein was concentrated by ultrafiltration with 10 kDa cut-off filter and analyzed by SDS-PAGE. Samples were prepared with 10% D_2_O (v/v) yielding concentration of 0.25 mM.

### NMR experiments

All NMR data were collected at 298 K on a Bruker Avance Neo 700 MHz and a Bruker Avance III 850 MHz spectrometers equipped with triple-resonance cryogenic probes. Sequential backbone assignments were obtained using Transverse relaxation-optimized spectroscopy (TROSY) versions (Pervushin et al. 1997) of conventional three-dimensional experiments (HNCO, HN(CA)CO, HNCA, HN(CO)CA, HN(CO)CACB, HNCACB and HN(CA)CB) (Sattler et al. 1999). IVL methyl group assignments were obtained using a two-dimensional constant time (CT) ^1^H-^13^C_methyl_ HMQC (Tugarinov and Kay 2003) and three-dimensional HMCMC, HMCM(C)CB, HMCM(CC)CA and HMCM(CCC)CO (Sinha et al. 2013). Additionally, a 3D 13Ch-13CH3 SOFAST-HMQC-NOESY-HMQC with a mixing time of 200 ms (Rossi et al. 2016), a 3D 15N-resolved NOESY with a mixing time of 400 ms, and a 3D 13C-resolved NOESY with a mixing time of 400 ms were used. All methyl assignment experiments were recorded with non-uniform sampling (NUS). NMR data were processed with Topspin 3.2 (Bruker), or with NMRPipe (Delaglio et al. 1995) and SMILE (Ying et al. 2017) and analyzed with Sparky (Lee et al. 2015).

### Assignment and data deposition

Spectra of *Tr*Bgl2 were initially assessed in a phosphate-based buffer at pH 6.0 and a TRIS-based buffer at pH 8.0. Spectral quality was markedly improved in TRIS buffer at pH 8.0, with several additional peaks becoming visible (data not shown). This observation, together the fact that a TRIS molecule was found at the active site of the crystallographic structure (Jeng et al. 2011), suggests that TRIS might stabilize apo *Tr*Bgl2.

**Fig. 1:**
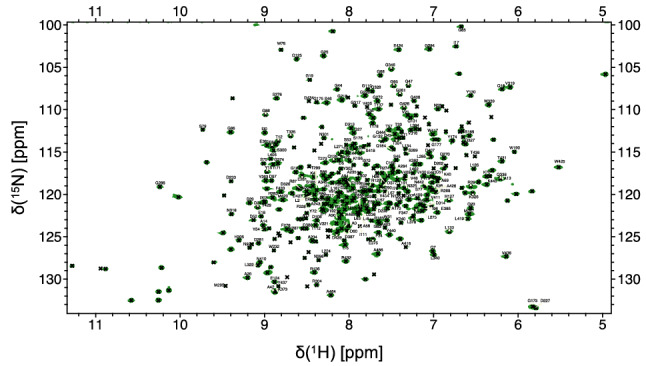
The assigned 700 MHz [^15^N,^1^H] TROSY NMR spectrum of *Tr*Bgl2. Assigned backbone N–H cross peaks are labelled with the corresponding residue number

**Fig. 2:**
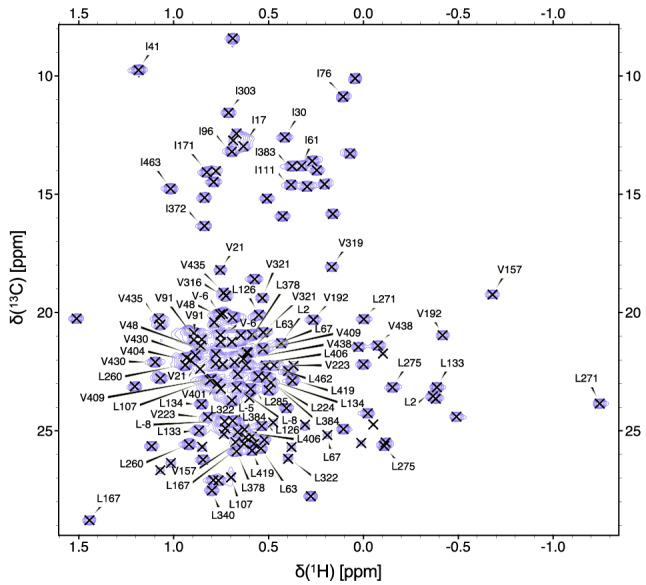
The assigned 700 MHz [^13^C,^1^H] HSQC spectrum of an IVL methyl protonated sample of *Tr*Bgl2. Methyl side chain resonances are indicated by the one-letter amino acid code and the sequence number is shown in black

The chemical shift assignment of *Tr*Bgl2 was performed in semi-automatic mode using the FLYA algorithm (Schmidt and Guntert 2012). The FLYA assignments were manually inspected and extended. Recombinant *Tr*Bgl2 consists of 488 amino acids (including an N-terminal His_6_ cleavable tag) and has a molecular weight of 55 kDa. In total, we were able to assign 265 backbone amide resonances and 55% of the methyl group resonances of the IVL residues, from 459 non-proline residues (Figs. 1 and 2). Assignment completeness is limited possibly due to incomplete back-exchange of perdeuterated amide groups, with only ~ 75% of the expected resonances being observed in the [^15^N,^1^H] TROSY spectrum of perdeuterated *Tr*Bgl2. Nonetheless, the assignments here reported provide a useful NMR basis for studies of the interaction of *Tr*Bgl2 with small molecules. The assignment of chemical shifts of *Tr*Bgl2 has been deposited into BMRB (https://www.bmrb.wisc.edu/) with accession number 50158.

**Fig. 3:**
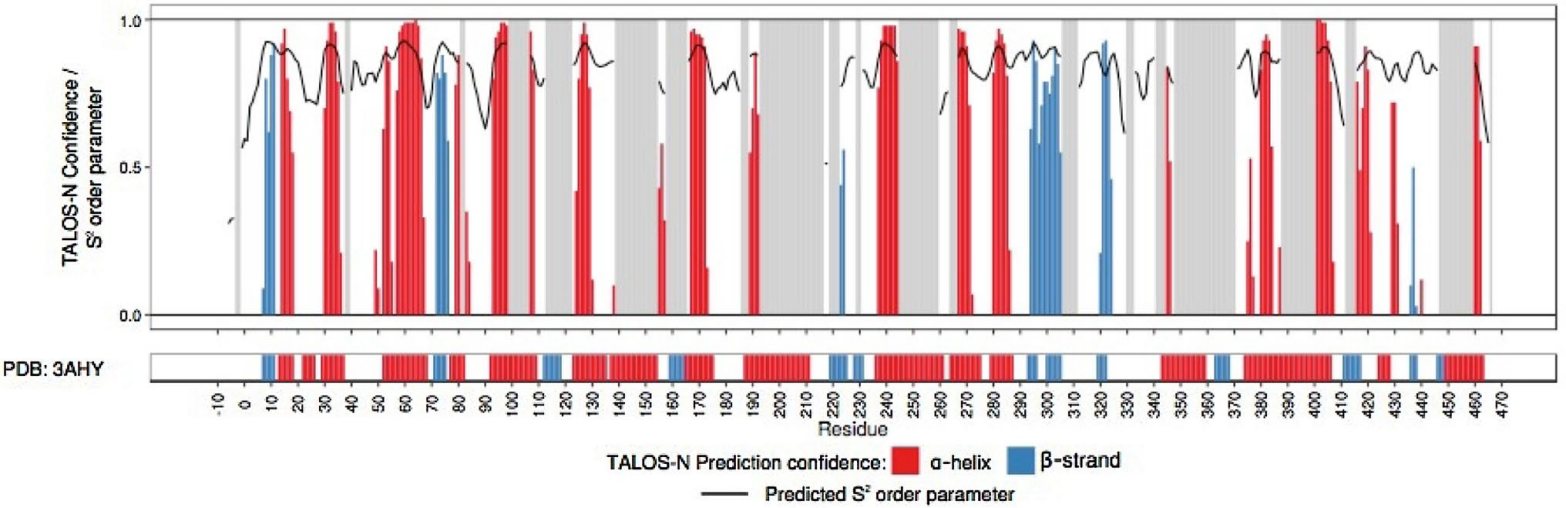
Secondary structure prediction of *Tr*Bgl2 analyzed with TALOS-N using the assigned chemical shifts compared to the secondary structure of the X-ray structure of *Tr*Bgl2. Top: colored bars (red and blue bars indicate α-helix and β-strands respectively) show the secondary structure type predicted by TALOS-N. The bar height represents the prediction confidence. The black line shows the predicted S^2 order parameter, a measure of flexibility. Bottom: the secondary structure of *Tr*Bgl2 as determined by X-ray crystallography (PDB ID 3AHY) (Jeng et al. 2011)

*Tr*Bgl2 secondary structure motifs were predicted by the TALOS-N software (Shen and Bax 2013) using the assigned backbone resonances as input data. The software indicated that the overall secondary structures are in agreement with the X-ray structure of *Tr*Bgl2 (PDB ID 3AHY) (Jeng et al. 2011) (Fig. 3).

## Data Availability

The data that support the findings of this work are available on BMRB with the following entry assigned accession number: 50158.
