# Correction to: ^1^H, ^13^C, ^15^N backbone and IVL methyl group resonance assignment of the fungal β-glucosidase from *Trichoderma reesei*

**DOI:** 10.1007/s12104-020-09967-2

**Published:** 2020-07-11

**Authors:** Eleni Makraki, Marta G. Carneiro, Alex Heyam, Eiso AB, Gregg Siegal, Roderick E. Hubbard

**Affiliations:** 1 YSBL, Department of Chemistry, University of York, Heslington, York UK; 2 ZoBio BV, J.H. Oortweg 19, 2333 CH Leiden, The Netherlands; 3 Division of Medicinal Chemistry, Amsterdam Institute for Molecules, Medicines and Systems (AIMMS), Vrije Universiteit, De Boelelaan 1108, 1081 HZ Amsterdam, The Netherlands; 4 Vernalis Research, Granta Park, Abington, Cambridge, UK

## Correction to: Biomolecular NMR Assignments 10.1007/s12104-020-09959-2

In the original publication of the article, the name of one of the authors is incorrect. The author's name is Eiso AB, but was modified to A. B. Eiso. The correct name is given in this Correction.

